# Narrowing farmland biodiversity knowledge gaps with Digital Agriculture

**DOI:** 10.1038/s44264-025-00118-5

**Published:** 2026-01-31

**Authors:** Ruben Remelgado, Michael Beckmann, Moudrý Vítězslav, Elisa Padulosi, Michela Perrone, Petteri Vihervaara, Christopher Marrs, Anette Eltner, Duccio Rocchini, Anna F. Cord

**Affiliations:** 1 Agro-Ecological Modeling Group, Institute of Crop Science and Resource Conservation (INRES), University of Bonn, Niebuhrstraße 1a, Bonn, 53113 Germany; 2 Chair of Environmental Planning, Brandenburg University of Technology, Erich-Weinert-Str. 1, Cottbus, 03046 Germany; 3 Department of Spatial Sciences, Faculty of Environmental Sciences, Czech University of Life Sciences, Kamýcká 129, Prague, 16500 Praha-Suchdol Czech Republic; 4 Department of Spatial Sciences, Faculty of Environmental Sciences, Czech University of Life, Kamýcká 129, Prague, 16500 Praha-Suchdol Czech Republic; 5 Faculty of Agricultural, Environmental and Food Sciences, Free University of Bolzano/Bozen, Piazza Universitá / Universitätsplatz 1, Bolzano/Bozen, 39100 Italy; 6 Nature Solutions, Finnish Environment Institute (SYKE), Latokartanonkaari 11, Helsinki, 00790 Finland; 7 Institute of Photogrammetry and Remote Sensing, Dresden University of Technology, Helmholtzstr. 10, Dresden, 01069 Germany; 8 BIOME Lab, Department of Biological, Geological and Environmental Sciences, Alma Mater Studiorum University of Bologna, Via Irnerio 42, Bologna, 40126 Italy

**Keywords:** Biodiversity, Agroecology, Sustainability

## Abstract

Digital Agriculture – broadly defined as the use of digital technologies and data to manage and optimize agricultural production systems – holds significant but largely untapped potential for biodiversity monitoring. Both fields share many (semi-)automated data collection technologies, analytical methods and workflows, but remain largely disconnected — and are sometimes even perceived as incompatible — in research, education and practice. Here, we explore how existing data streams from Digital Agriculture can directly contribute with primary biodiversity data required by policy-relevant applications, linking them to the Essential Biodiversity Variables framework. We discuss the benefits of this integration, its challenges, and outline pathways for its adoption with respect to ongoing advances in biodiversity science and policy. This integration could improve the precision of biodiversity conservation in farmland, and accelerate transitions to sustainable agriculture – an urgent priority to safeguard nature and its contribution to people.

## Introduction

**Fig. 1:**
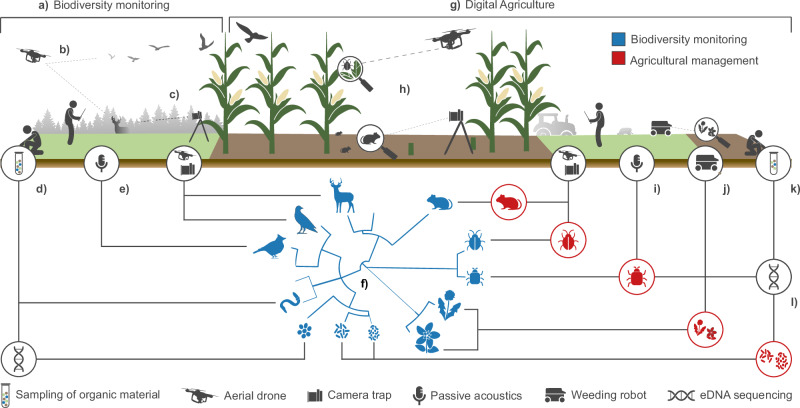
Uncovering biodiversity through Digital Agriculture. Biodiversity monitoring (**a**) is supported by various technologies, including drones (**b**), camera traps (**c**), eDNA obtained from genetic samples (**d**), and passive acoustic monitoring (**e**). These technologies record species occurrences and partly abundances, enabling subsequent assessments of species distributions and community compositions (in blue; **f**). Digital Agriculture (**g**) uses the same technologies in routine field and farm management (shown in red). Drones and camera traps (**h**), sometimes combined with passive acoustic monitoring (**i**), monitor pest occurrences and damages. Simultaneously, weeding robots facilitate the detection and removal of weeds (**j**) that compete with crops for space and nutrients. Soil sampling (**k**), often used to control for nutrient availability, may also support the extraction of eDNA to control for harmful pathogens (**l**). Because biodiversity monitoring (**a**) shares technology requirements with Digital Agriculture (**g**), data from the latter, if made accessible to biodiversity monitoring experts, could improve assessments of species populations and community composition (in blue; **f**).

Both agriculture and biodiversity science are undergoing rapid digital transformations. Research fields that were once constrained by sparse, irregular, or coarse-resolution data are now becoming increasingly data-rich. In agriculture, the widespread adoption of automated sensors and digital technologies for precision farming (hereafter ‘Digital Agriculture’) — including cameras^1^, drones^2^, and genetic sampling^3,4^ enables continuous, high-resolution monitoring^5,6^. For instance, drones now support a wide range of tasks, such as sowing of seeds, spreading fertilizers and feeds, counting livestock, and the monitoring of crop health and yield^7^. These data are used to maximize yields, reduce food waste, minimize inputs, and increase the resilience and sustainability of farming systems^8^. Biodiversity monitoring is being revolutionized by the same sensing technologies^9–11^, which use virtually the same types of data, and similar sensor networks, for unprecedented species- and community-level biodiversity assessments^12,13^. This convergence in technologies creates a unique opportunity: to align agricultural and ecological monitoring infrastructures, methods, and objectives. Through digital agriculture, sensors established for digital field and farm management could also generate primary biodiversity data required for biodiversity monitoring and policy-relevant reporting^14,15^. In fact, both applications may even employ the same analytical methods (e.g., same image segmentation algorithms^16,17^), increasing the potential for interoperability. Notably, some primary biodiversity data are already generated through Digital Agriculture (Fig. 1), though collected for purposes other than biodiversity monitoring (e.g., pest control^1,18,19^, soil management^20–22^, crop phenotyping^23–25^). In some cases, primary biodiversity data may even be collected unintentionally and treated as ‘noise’ (e.g., molehills in drone imagery^26^).

A growing body of literature recognizes the potential of Digital Agriculture for biodiversity. For instance, farm management data and information systems have been proposed as conservation tools by identifying low-productivity zones suitable for conservation strips^6^. At the policy-science interface, there is a growing push for the widespread adoption of Digital Agriculture. The 2022–2031 strategic plan of the Food and Agriculture Organization (FAO), for example, promotes the integration of digital technologies to optimize the use of natural resources, reduce environmental impacts, and encourage biodiversity-friendly farming methods^27^. This aligns with the Global Biodiversity Framework (GBF) targets, particularly those related to sustainable land management (Target 10), reduced food waste (Target 16), and lower agricultural pollution (Target 7). However, despite these overlapping objectives, biodiversity monitoring initiatives largely operate independently of the rapidly evolving Digital Agriculture sector. This disconnect limits the integration of high-resolution, near real-time agricultural and biodiversity data, potentially missing valuable opportunities for more precise and adaptive conservation actions.

Indeed, such data integration remains rare among state-of-the-art agroecological monitoring initiatives. While meta-analyses have attempted to link yield and biodiversity measured at the same locations^28,29^, many biodiversity studies still rely on (sub)national agricultural statistics to assess biodiversity-yield interactions^30,31^. This reliance often stems from a lack of direct and spatially representative measurements of crop production in areas where biodiversity is surveyed, for instance, due to land access restrictions^32^. When aggregated statistics are used, assessments of biodiversity change inherit temporal inconsistencies and quality issues^33^, which may additionally lack ecologically meaningful thematic and spatial detail^34^. Because biodiversity data frequently originate from short-term or taxonomically narrow monitoring efforts^35–37^, aligning biodiversity and crop production data becomes increasingly challenging. Data misalignments and biases can influence the conclusions of biodiversity change assessments^38^, resulting in biased perceptions of biodiversity responses to agricultural management practices.

Ensuring the robustness of biodiversity change assessments is critical. Policy frameworks for protecting and restoring nature (e.g., GBF, EU Nature Restoration Regulation) rely on these measurements—and on the inference of change drivers—to assess progress towards biodiversity targets^39^. In agroecosystems, monitoring species occurrences, abundances, and traits is especially important, as their combination informs on variations in the provision and stability of nature’s contributions to people (e.g., pollination, pest control), which in turn influence human well-being and food security^40^. To effectively capture functional changes in agroecosystems, biodiversity monitoring must be systematic and accompanied by field- and farm-level management data. Such integration allows improved understanding of temporal variations in biodiversity responses to the sowing, growth, and harvest cycles of crops^41,42^, and to the sensitivity of those responses to local environmental and socio-economic context^35^.

In this paper, we discuss how Digital Agriculture can meet these requirements, enabling more systematic and concurrent measurements on biodiversity and agricultural production. First, based on existing literature, we demonstrate that biodiversity monitoring can, in principle, already be enabled by common applications in Digital Agriculture. Second, we map core data streams to specific, policy-relevant indicators on the status and change of biodiversity. Third, we propose pathways to improve the operationalization of Digital Agriculture for monitoring biodiversity. Finally, we reflect on ongoing investments in the digitalization of agroecosystems, and discuss the key challenges, limitations and implications of this approach.

## Opportunities for monitoring biodiversity through Ddigital Agriculture

Digital Agriculture technologies are primarily designed to monitor variables within cultivated lands, particularly croplands. Therefore, their value for biodiversity monitoring is limited to the collection of data on taxa occurring within those lands. However, despite the fact that semi-natural habitats are key for sustaining farmland biodiversity^43^, production fields can host large portions of known species pools (e.g., up to 51% of farmland vascular plants, earthworms, spiders, and wild bees in Europe^44^). These species are commonly affected by—and adapted to—agriculture-related habitat transformations^45,46^, driving temporal and spatial variations in biodiversity patterns^41,42^. Data on the cropland-dwelling fraction of biodiversity may already be generated through Digital Agriculture (Fig. 1). Here, we exemplify this potential through three, common applications of Digital Agriculture generating relevant, yet underused, data on species occurrences and traits: the control of pests, diseases, and weeds (**2.1**), the monitoring of soil nutrients and pathogens (**2.2**), and the phenotyping of domesticated plants (**2.3**).

### Management of pests, diseases, and weeds

Globally, pests and crop diseases cause an estimated 20-40% loss in food production each year, amounting to approximately US$220 billion in damages^47^. The most damaging pests include invasive mammals, insects, and birds^48,49^. Weeds further contribute to crop losses^50^ by competing with crops for water, nutrients and space as well as providing habitat for insect pests, and potentially harboring harmful pathogens^51^.

The monitoring and management of pests, diseases, and weeds is a key application of Digital Agriculture, guiding the use of pesticides^19^, weed removal^52^, and deterrents (e.g., audio playbacks of animal calls to repel competing species^53^). Drones equipped with RGB, multi- or hyperspectral sensors, or stationary cameras help detect crop damages caused by insects ^54^, rodents^55^ or larger mammals^56^. Alternatively, traps and acoustic sensors can be used to locate pests (e.g., insects^57^, birds^18^) and can potentially inform on their seasonal occurrence and abundance (i.e., phenology)^58^. Drones, sometimes combining RGB and lidar sensors, may also be used for detecting weeds^59^ and plant disease^2^.

Biodiversity surveys often rely on the same data and technologies. Drones have long been used for this purpose^11^, enhancing estimates of species richness and abundances (e.g., for birds^60^ or endangered and elusive mammals^61^). Similarly, camera traps are commonly used to detect various taxa^9,62^—although rarely in agricultural lands^63^—as are camera-equipped insect traps^64^ and passive acoustic sensors^65^. The generated imagery and audio recordings may then be analyzed with AI-based models to identify and distinguish species^66–69^.

### Monitoring of soil nutrients and pathogens

Poor soil conditions are a critical constraint on food production globally. The United Nations estimate that up to 40% of the world’s land is degraded, affecting close to 3.2 billion people^70^. Soil salinity alone, which hinders crop productivity^71^, has led to reductions in agricultural yields of up to 70% in the affected areas^72^. The global costs of land degradation are substantial, estimated at US$ 878 billion per year, with regard to agricultural productivity, nature’s contributions to people, and other related sectors^70^.

Periodic assessments of soil health are essential for optimizing crop productivity, and thus a key application of Digital Agriculture. Soil sampling at the field-level shows variations in mineral compositions^73^, nutrient contents^74,75^, and functionality^20^. In this process, the abundance of specific microorganisms may be monitored as a proxy for soil quality (e.g., earthworms^76^). More recently, thanks to advanced genetic testing, the use of environmental DNA (eDNA) has become increasingly affordable and efficient^22^, enabling detections of root-level microbial communities affecting plant growth^3,22,74^. Data collected on the field can then be combined with imagery from drones or satellites to map soil quality indicator^77^. This can guide site-specific recommendations for use of fertilizers^21^ and pesticides^78^, and of crop rotation schedules^79^.

The monitoring of soil biodiversity relies on similar inputs. eDNA, in particular, has been shown effective in measuring soil biodiversity^10^, and may also inform on occurrences on unobserved—and potentially elusive—species that transverse sampled locations^80^. The use of eDNA for biodiversity monitoring may, in the future, also integrate drone and satellite imagery to map the diversity of soil-dwelling bacteria and fungi beyond sampled locations^81^.

### Phenotyping of domesticated species

Over the past three decades, approximately $3.8 trillion in crop and livestock production has been lost due to disasters^82^. These events have contributed to increased food prices, a trend expected to continue under climate change^83^. This has major implications for food security, putting millions of people at risk of malnutrition.

Phenotyping, which implies measuring and analyzing structural, physiological, and biochemical characteristics (i.e., traits) of plants and animals, can help tackle these issues. Digital agriculture systems may use phenotyping as part of routine management of crops throughout their lifecycle, optimizing the spatial and temporal allocation of natural and chemical resources (e.g., through targeted irrigation^84^). This can be enabled through the use of multi- and hyperspectral or lidar imagery, derived from drones or satellites, to track potential growth deficiencies revealed by, for instance, low carbon concentrations^85^ or spatial variations in the vertical structure of plant stands^86^. Drone imagery may also be used to monitor livestock health, such as by informing on the body mass, temperature, or feed intake of individual animals^87^.

Trait-based approaches are increasingly used in biodiversity monitoring. This enables data to inform on the response of organisms to environmental change, species adaptation patterns, and overall population or ecosystem health. In this process, imagery from drones is mostly used to record functional traits of plant communities^88^, including wild plants occurring in agricultural fields^16^. More recently, as in livestock management applications, drones have been used to infer traits of wild mammals, such as body mass^89^.

## Digital Agriculture As Vehicle For Sustainable Transitions

**Fig. 2:**
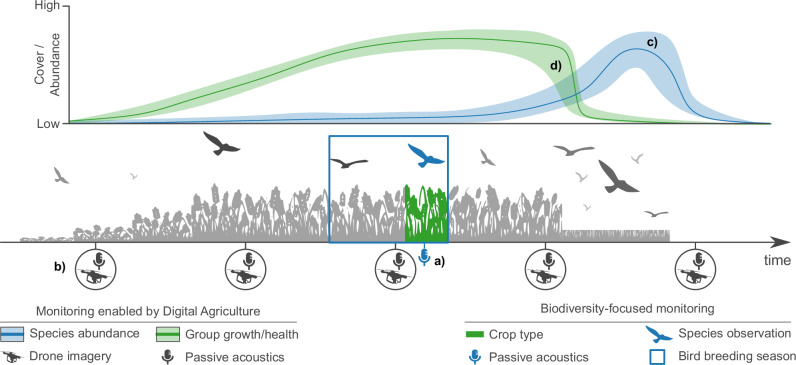
Potential for farm- and field-level causal inference. **a** In a biodiversity-focused survey, species observations (bird icon, in blue) are detected with passive acoustic monitoring. The timing of the survey is aligned with the main breeding season of the target species (blue outline). Species detections are paired with categorical information on crop type (in dark green), common practice in state-of-the-art agroecological research. **b** Passive acoustic sensors are used to detect crop-damaging birds, while drones help monitor crop growth, damages, and health, but could also enable time-series on species-level bird abundances (**c**) based on detections recorded outside the breeding season (bird icons, in black). **d** With time-series on agriculture and biodiversity, together with their uncertainties (shaded ribbons), we can formally measure the presence and magnitude of causal effects, such as relating to biodiversity-yield or predator-prey interactions.

Biodiversity monitoring through Digital Agriculture would ideally support a technological-ecological transition from output-optimizing (focused on crop growth and health) to ecosystem condition-optimizing. It would thus allow a transition from reactive to proactive biodiversity management as real-time and spatially explicit data would enable continuous monitoring of species and ecosystem health, making biodiversity protection an integral, ongoing part of field and farm management (Fig. 2).

Through Digital Agriculture, cultivated lands, which now comprise nearly half of the world’s habitable land^90^, may become “living labs” where key biodiversity data are regularly collected. Time-series on species distributions and abundances, combined with those on crop growth and agronomic management, may be used to infer dynamics in ecological responses to environmental change^91^ potentially overlooked by structured, yet seasonal and temporally limited, biodiversity surveys. This would facilitate new insights into ecological dynamics related to movement phenology^42^ and habitat use in agricultural land for foraging^46^, predation^92^, or reproduction^93^. If data from multiple farms is integrated through state-of-the-art sampling designs^94^, Digital Agriculture could help sustain scalable causal inference frameworks essential for policy-relevant biodiversity change reporting^95^ and evidence-based agricultural management^96,97^.

To enable insights relevant to both policy-making and reporting, Digital Agriculture data can be aligned with the Essential Biodiversity Variables (EBVs) framework by the Group on Earth Observation Biodiversity Observation Network (GEO BON)^98^. Long endorsed by the biodiversity research community^98–100^, EBVs were proposed as a means of standardizing and streamlining biodiversity monitoring and reporting across scales^98^, and now form the basis for headline indicator 21.1 of the GBF^101^. Many EBVs require species-focused reference data for their calculation and calibration, and much of these data can be derived by digital technologies used in both biodiversity monitoring and Digital Agriculture^102,103^—as described in section 2.

**Fig. 3:**
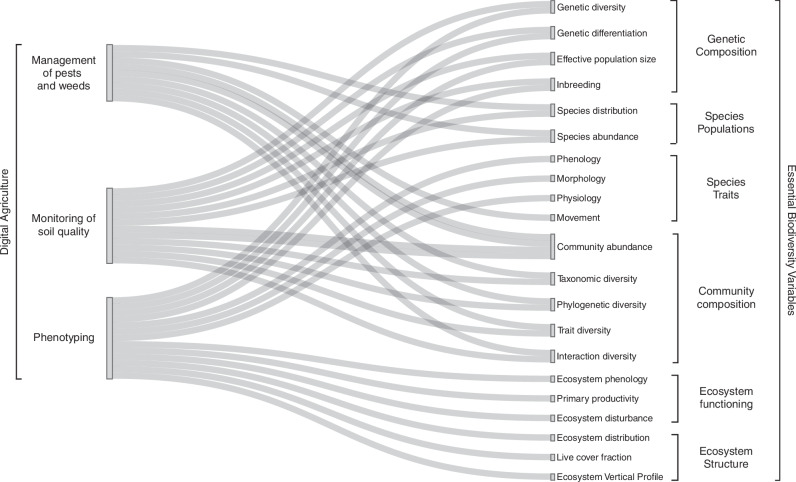
Potential synergies between Digital Agriculture and EBVs. On the left, the different applications of Digital Agriculture highlighted in this paper, namely pest, disease and weed control, soil monitoring, and phenotyping. On the right, Essential Biodiversity Variables (EBVs) per thematic group. Lines link each Digital Agriculture application with one or multiple EBVs. A detailed description on each EBV, their relevance for agriculture, and their enabling Digital Agriculture Technologies is given in Supplementary Table 1. The data used in this figured is provided as Supplementary Data 1.

Digital Agriculture therefore can enable these data streams, thereby supporting direct (or indirect) calculations of several EBVs (Fig. 3, Supplementary Table 1). For example, species observations derived through the monitoring of pests, weeds, diseases, and soil health can be used directly to calculate EBVs on species abundances. Similarly, phenotyping data can inform EBVs on species-level traits, for example morphological (e.g., plant height, body size of insects) physiological (e.g., nutrient content, body temperature), and production-related traits (e.g. number of flowers or nests). Time series of these EBVs, enabled through the regular, systematic monitoring of crop growth and of associated biodiversity, could further support assessment of EBVs on the phenology of single species, and community-level variations of trait, taxonomic, phylogenetic, and interaction diversity. If monitoring capabilities are integrated at landscape level, EBVs on species movements and distributions may be inferred (e.g., using networks of cameras^9^, acoustic sensors^104^). Concurrent data on the growth of crops could further inform EBVs on ecosystem function and structure. Specifically, phenotyping data and management could enable EBVs on the vertical profile, live cover fraction, and primary productivity of agroecosystems, and on their disturbance regimes (e.g., relating to sowing, in-season management and harvesting).

## Challenges And Enablers Of Biodiversity Monitoring Through Digital Agriculture

National biodiversity monitoring schemes are required to guide conservation action^105^. Yet, such programs remain absent in most countries, or largely underdeveloped or underfunded^105^. This may be explained by the costs of such programs, reaching millions of US$ per year^106,107^. While such investments are feasible for most countries of the Global North^106,107^, in the Global South, global disparities in economic capacity and institutional stability persist^108^. Investments in Digital Agriculture could help balance the organizational and financial burdens of biodiversity monitoring in agroecosystems, enabling the monitoring of emergent conservation issues (e.g., human-wildlife conflicts due to cropland expansion^92^).

**Fig. 4:**
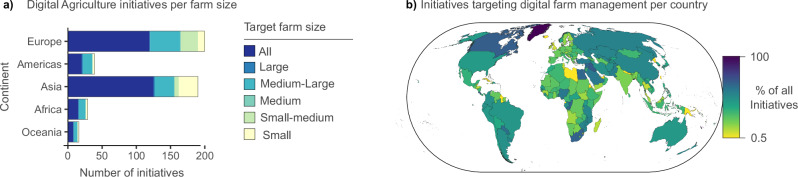
Global distribution of initiatives advancing Digital Agriculture. **a** For each continent, the number of ongoing Digital Agriculture initiatives recorded by the FAO^109^ that target farm-level management. Colors distinguish initiatives based on the farm size they target, with sizes classified according to FAO’s Agritech Observatory^109^: “Small” - typical characteristics associated with “smallholder” farms, such as limited access to resources, finance, and technology; “Medium” - highly active, specialized farms and agricultural holdings; “Large” - industrial farming. **b** For each country, the proportion of all ongoing Digital Agriculture initiatives targeting farm-level management. This includes sensing technologies, and digital platforms for engaging with field- and farm-level data in decision making. See Supplementary Data 2 to access the plotted data.

Indeed, investments in Digital Agriculture are accelerating. As of January 2025, the FAO recorded 449 ongoing, public or private initiatives aimed at advancing the use of Digital Agriculture across all continents^109^, ~89% of which are applied to managing farms of all sizes (Fig. 4a). While ~90% of all initiatives concentrate in Europe and Asia (Fig. 4b), every country hosts at least some initiatives that apply Digital Agriculture using automated sensors, Big Data and Artificial Intelligence^109^. In the Global South, most private investments in the agricultural sector are already directed at digital innovation^110^, notably in countries such as Thailand and Vietnam, where the density of drones per km² of cropland ranks amongst the highest^7^. These current and future investments in Digital Agriculture hold great potential to enhance national biodiversity monitoring capabilities, provided that digitalization efforts *i*) tackle inequities in digitalization capacity, *ii*) provide direct benefits for farmers, *iii*) enable data sharing and protect data privacy, and *iv*) directly account for data gaps, biases, and quality issues. In this section, we discuss these aspects and pathways to enable them.

### Equitable and inclusive digitalization to mitigate socio-economic risks

While investments in Digital Agriculture are increasing globally, digitalization rates still vary greatly across farming systems. The development of new technologies tends to favor large, capital-intensive farms, which can then quickly adopt new technologies^111^, while smallholder and family farms, despite largely welcoming digitalization^112–114^, often lack the required technical support^112^ and policy incentives^115^. Recent data on the global diffusion of agricultural drone technologies indicate that, beyond income levels, factors, such as labor scarcity, the age structure of the farming population, subsidies, and regulatory frameworks also play key roles^7^.

For instance, in South Africa, smallholder farmers struggle with high labor and maintenance costs and weak public infrastructure^116^. In Ghana, digitalization improved farmer organization into groups, but resources remain insufficient to enhance overall working condition^117^. Without adequate political and expert support, smaller farms may struggle to compete economically, exacerbating existing socioeconomic inequalities. In addition, if digitalization efforts do not succeed in smallholder- and family-owned lands, which compose over 90% of the world’s farms^118^, Digital Agriculture may exacerbate the spatial, temporal, and taxonomic biases affecting global biodiversity data^119^.

Ongoing development programs can help guide future digitalization efforts. For instance, in the Global North, the European Agricultural Fund for Rural Development (EAFRD) reserved €8 billion between 2021 and 2027 to foster the digital transformation of rural communities, with particular focus on family farms^120^. In the Global South, national incentive schemes are gradually emerging^121,122^. These are supported by international development programs (e.g., UN-led *50 by 2030* program^123^), some aiming at developing monitoring capabilities in rural areas (e.g., FAO’s AGRI-NBSAP^124^). However, the effectiveness of such programs rests on the existence of inclusive policies and region-sensitive governance^7,125,126^. Digitalization efforts must consider the socio-economic and environmental contexts of rural communities^127,128^. Context-aware digitalization initiatives ease the adoption of new technologies by resource-constrained smallholder farms^129^, and create new employment opportunities^7^.

### Achieving shared benefits through digitalization

**Fig. 5:**
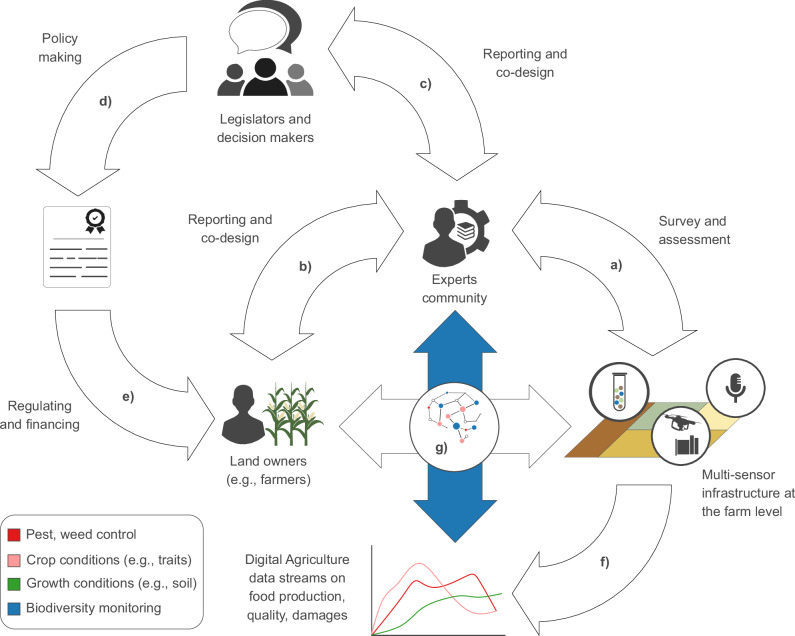
Farmer participation strategy. In current participatory strategies, expert communities conduct research on interactions between biodiversity and field management practices (**a**), and may involve farmers in monitoring activities and data analyses (**b**). Resulting literature fuels science-based reporting (**c**). Policymakers use these reports as a basis for legislation (**d**) aimed at regulating and funding field-level, biodiversity-supporting management practices (**e**). In this process, farmers profit from biodiversity monitoring only indirectly. Yet, they are exposed to the uncertainties of biodiversity gains, and of their influence on food production. Biodiversity monitoring, if established through Digital Agriculture, could directly provide insights on agroecosystem conditions and trends (**f**). Resulting Digital Agriculture data streams would then offer insights on trade-offs between food production and biodiversity (**g**).

Humans remain central to transition towards sustainable agriculture^130,131^, with farmers ultimately responsible for deciding on uptake of digital technologies, for implementing agri-environmental measures^126^, and for giving access to farmlands for biodiversity data collection. To address this issue, a range of participatory strategies have been proposed^132^, some connecting networks of stakeholders (including farmers), data, tools, and biodiversity monitoring programs^105^ (Fig. 5a–e**)**. Modern participatory approaches aim to incorporate not only ecological objectives, but also cultural, economic, and societal considerations^105^ (Fig. 5a, b). Yet, current participatory strategies can impose a considerable burden on farmers. While these strategies may involve farmers in the collection, analysis and reporting of data-driven insights (Fig. 5c–e), this often constitutes a challenging, and time-consuming task, adding to the challenges of farming itself. Simultaneously, despite the vast literature on the biodiversity benefits of agroecological measures, the direct benefits for individual farmers remain uncertain, and scale- and context-dependent^35^. As a result, generic policy recommendations or incentive schemes may fall short, and may inadvertently fuel negative human perspectives on biodiversity conservation^133^.

Digital Agriculture may help streamline the involvement of farmers in biodiversity monitoring, as it offers direct, actionable insights about field- and farm-level environmental conditions, enhancing everyday agricultural management (Fig. 5g). In this process, targeted, long-term biodiversity gains could be achieved through state-dependent adjustments of farm management practices to biodiversity observations, potentially also supported through agri-environmental schemes^134^. To enable biodiversity monitoring through farm-level digitalization, projects advancing biodiversity monitoring capabilities (GBF Target 21) could sustain cooperation projects designing, or optimizing, farm-level sensor networks. Indeed, such developments would align with the GBF, which calls for capacity-building, technology transfer, and cooperation in biodiversity monitoring (Target 20). Finally, economic benefits of Digital Agriculture may sustain biodiversity monitoring capabilities beyond a project’s lifespan.

### Data ownership and privacy concerns

Digital Agriculture will generate vast quantities of environmental and field-to-farm-level management data, with important questions arising on who owns, controls, and benefits from these data. Over 70% of open data on agriculture originates from satellite-based remote sensing applications^135^. On the contrary, farm-level data remain largely unavailable and lacking in interoperability^112^.

Ongoing initiatives are addressing data access limitations. Global programs (e.g., UN-funded CGIAR^136^), nationally-funded expert groups (e.g., FAIRagro in Germany^137^), and multi-national data harmonization projects^138,139^ are rapidly aiming to make farm-level data Findable, Accessible, Interoperable, and Reusable (FAIR). As these initiatives advance, Digital Agriculture data streams can become accessible for biodiversity monitoring. This will require clear and transparent data-sharing agreements to protect data property and rights (GBF targets 21, 22) and to avoid potential misuse of information or surveillance-like dynamics^140^. This is critical, given that economic details are involved, such as subsidies linked to participation in agri-environmental schemes. Volunteer and federated data access, as adopted by the Global Biodiversity Information Facility (GBIF), would help preserve data ownership and privacy. Alternatively, data sharing formats may directly reflect national regulations and privacy agreements through custom spatial aggregations^141^.

However, it is critical that data provision is assured to support reporting requirements. If data are treated exclusively as private property, they may become a commodity reserved for activities with higher financial benefits compared to biodiversity monitoring^7^. In farmland, this may occur with respect to companies producing and renting machinery, who might impose restrictions over the access of data generated by tractors and other sensors. In Europe, the EU Data Act is setting an important example in tackling this issue, empowering farmers to access and share data from connected farm equipment^142^. This gives the managers of farmland control over their operational data, giving users rights to access this data, while also creating obligations for businesses that provide these products.

### Integrating ecological knowledge to address data biases

Georeferenced information on the occurrence and abundance of species derived through Digital Agriculture cannot replace structured, long-term data streams required for biodiversity trend reporting. However, it may complement them, helping mitigate pervasive spatial and temporal data gaps and biases in cultivated lands^32^. For instance, FAIR principles for sharing whole-genome sequencing data obtained from soils^143^ could inform on soil-dwelling species, tackling taxonomic biases in global biodiversity databases^144^. Digital Agriculture may also reveal small-bodied species undetected during biodiversity surveys, or when land-access restrictions are in place (e.g., nests of ground-breeding birds^145^).

However, Digital Agriculture alone cannot resolve existing data biases. In addition to the taxonomic and body-size-related biases discussed above, its benefits may vary per planted field and be conditioned by crop types and associated management practices. For instance, seasonal and diurnal variations in species distributions may be misaligned with crop growth periods. Biodiversity observations resulting from Digital Agriculture may further be limited in scope, such as targeting the most harmful pest species. Because such biases are very familiar to biodiversity surveyors^36^, their involvement (e.g., guidance on sensor selection and data collection periods) may enhance the usability of biodiversity data obtained through Digital Agriculture for downstream analyses. This may require improvements in the governance of on-going monitoring schemes and a better involvement of stakeholders^146^.

## Conclusions

Biodiversity conservation efforts are gaining momentum through international policy, such as the GBF or the EU Nature Restoration Regulation. Many targets outlined in the GBF depend on sustainable farm management^147^, which in turn requires concerted monitoring efforts to track biodiversity trends. Digital Agriculture offers a promising, cost-effective pathway to bridge agricultural and biodiversity monitoring. In fact, it employs some of the same technologies and methods used to monitor biodiversity, making derived data—which already captures species occurrences and traits—usable for concurrent biodiversity monitoring, scientific discovery, and learning. Most importantly, this would be achieved without placing additional burdens on farmers, with direct benefits for them. Yet, the monitoring of agriculture and biodiversity remain disconnected in research, practice and education. Explicit integration is needed to minimize monitoring costs and pave the road towards more sustainable future farming systems.

## Supplementary information

Supplementary.

SupplementaryData_1.

SupplementaryData_2.

## Data Availability

No data were collected for this study.
