# Supplementary material for: Narrowing farmland biodiversity knowledge gaps with Digital Agriculture
Source: NPJ Sustain Agric. 2026 Jan 31;4(1):10. doi: 10.1038/s44264-025-00118-5 (PMC12860687; doi:10.1038/s44264-025-00118-5)
Supplement: Supplementary file 1 — Supplementary. [file 44264_2025_118_MOESM1_ESM.pdf]

**Supplementary Table 1. Links between the EBV framework and Digital Agriculture.** Table describing how Digital Agriculture enables Essential Biodiversity Variables (EBVs) included in GEO BON's EBV framework. Each row describes a single EBV, distinguishing its thematic group, name, and description. (given by the columns "Group", "Name", and "Description", respectively). In addition, information is provided on how each EBV is contributes to agroecosystems (column "Relevance for agriculture"), as well enabling data and technologies (column "Potential DA data sources (incl. technologies, sensors, and variables)") used in the scope of Digital Agriculture.

| **Group** | **Name** | **Description** | **Relevance for agriculture** | **Potential DA data sources (incl. technologies, sensors, and variables)** |
|---|---|---|---|---|
| **Genetic composition** | Genetic diversity | Differences in DNA sequences among individuals within a single species | Identifies adaptive potential and resilience of crop and livestock populations'; informs breeding and phenotyping | Genomic sequencing, eDNA metabarcoding |
| | Genetic differentiation | Variation in allele identity and frequency distinguishing populations of the same species | Guides management of both cultivated and wild populations to maintain diversity | Spatially referenced genotyping |
| | Effective population size | size of an idealized population that would lose genetic diversity at the same rate as the real population | Indicates vulnerability to genetic drift or loss of diversity, supports long-term sustainability of crop/animal populations. | Genomic sampling over time, census proxies from drone/satellite counts |
| | Inbreeding | Reproduction between genetically-related individuals | Detects potential fitness loss and informs breeding programs to sustain population vitality | Genotyping |
| **Species populations** | Species distributions | Probability of a species occurring across space and time | Identifies key areas for conservation or pest control, enables integration with farm-level data for biodiversity-yield assessments | Drone imagery, GPS field surveys, citizen science apps |
| | Species abundances | Estimated number of individuals of a species occurring across space and time | Tracks population trends, enables early detection of pest outbreaks or declines in beneficial species; supports farmland biodiversity indicators (e.g. Farmland Bird Index) | Data from drone, camera trap, or acoustic sensors sourced by models deriving species counts. |
| **Community composition** | Community abundance | Total number of individuals across all species within ecological assemblages | Indicates community-level productivity and ecosystem health | Camera traps, drone imagery, weeding robots |
| | Taxonomic diversity | Diversity and identity of species within ecological assemblages | Identifies beneficial or harmful species within agricultural landscapes | |
| | Phylogenetic diversity | Evolutionary relationships and distinct lineages represented within ecological assemblages | Highlights evolutionary uniqueness and resilience of communities | |
| | Trait diversity | Range of functional traits expressed by organisms | Indicates the potential for ecosystem services such as | |

| | | | | |
|---|---|---|---|---|
| | | within ecological assemblages | pollination and nutrient cycling | |
| | Interaction diversity | Diversity and structure of interactions among organisms across trophic levels | Provides insights into ecological networks supporting pollination, pest and weed regulation, and soil quality | |
| **Ecosystem functioning** | Primary productivity | Rate at which energy is converted into organic matter through photosynthesis | Provides information on crop biomass and yield | NDVI/EVI time series from drone imagery |
| | Ecosystem phenology | Timing, duration, and intensity of cyclical ecological processes | Informs planting, harvesting, and monitoring of biodiversity-yield interactions | Drone imagery, phenocams |
| | Ecosystem disturbances | Sudden disruptions in ecosystem functioning that deviate from regular dynamics | Supports adaptive management against sudden changes (e.g., drought, pests) | Change detection from drone imagery |
| **Ecosystem structure** | Live cover fraction | Proportion of ground or substrate area occupied by living vegetation | Informs on the fractional cover of crops | Drone imagery |
| | Ecosystem distribution | Spatial distribution of discrete ecosystem units | Maps crop types, crop diversity, semi-natural landscape features and field size distribution | Land cover maps from satellite/drone imagery |
| | Ecosystem Vertical Profile | Vertical distribution (above and below the land surface) of biomass in ecosystems | Supports carbon assessment and soil quality monitoring, detects crop anomalies, e.g. in height structure | Drone imagery |

**Supplementary Data 1. Digital Agriculture data streams.** Data used to construct **Figure 3** of the main manuscript. Each Digital Agriculture application described in Section 2 of the main manuscript (column "source"), an Essential Biodiversity Variable (EBV) is given (column "target"), which can be supported by data generated by the source. Each EBV is then connected to a larger thematic group (column "target category") as per GEO BON's EBV framework.

**Supplementary Data 2. Global Digital Agriculture Initiatives.** Data used to construct each panel of Figure 4, which depicts the global distribution of Digital Agriculture initiatives per continent and country. The data was sourced from the AgriTech Observatory (https://agritechobservatory.fao.org/).
